# Green manure incorporation enhanced soil labile phosphorus and fruit tree growth

**DOI:** 10.3389/fpls.2024.1356224

**Published:** 2024-02-26

**Authors:** Yuanyu Yang, Jianwei Zhang, Xia Chang, Lunlun Chen, Yongmin Liu, Qingwei Xu, Mengjuan Wang, Haiyan Yu, Renmei Huang, Jie Zhang, Yingxiao Hu, Qijuan Hu, Xiaojun Shi, Yuting Zhang

**Affiliations:** ^1^ College of Resources and Environment, Southwest University, Chongqing, China; ^2^ Interdisciplinary Research Center for Agriculture Green Development in Yangtze River Basin, Southwest University, Chongqing, China

**Keywords:** orchard, phosphorus turnover, stable oxygen isotopes, microbial mobilization, cover crop

## Abstract

**Introduction:**

The incorporation of green manures substantially enhances the conversion of external phosphorus (P) fertilizers and soil-reserved P into forms readily available to plants. The study aims to evaluate the influence of green manure additions on soil phosphorus dynamics and citrus growth, considering different green manure species and initial soil phosphorus levels. Additionally, the research seeks to elucidate the microbiological mechanisms underlying the observed effects.

**Methods:**

A citrus pot experiment was conducted under both P-surplus (1.50 g·P·kg^-1^) and P-deficient (0.17 g·P·kg^-1^) soils with incorporating legume (Leg), non-legume (Non-Leg) or no green manure residues (CK), and ^18^O-P labeled KH_2_PO_4_ (0.5 g, containing 80‰ δ^18^O_p_) was additionally introduced to trace the turnover characteristics of chemical P fertilizer mediated by soil microorganisms.

**Results and discussion:**

In P-surplus soil, compared with the CK treatment, the Leg treatment significantly increased soil H_2_O-P_i_ (13.6%), NaHCO_3_-P_o_ (8.9%), NaOH-P_i_ (9.5%) and NaOH-P_o_ (30.0%) content. It also promoted rapid turnover of P sources into H_2_O-P_i_ and NaHCO_3_-P_i_ pools by enhancing the *phoC* (576.6%) gene abundance. In contrast, the Non-Leg treatment significantly augmented soil H_2_O-P_i_ (9.2%) and NaHCO_3_-P_o_ (8.5%) content, facilitating the turnover of P sources into NaHCO_3_-P_i_ pools. Under P-deficient soil conditions, compared with the CK treatment, the Leg treatment notably raised soil H_2_O-P_i_ (150.0%), NaHCO_3_-P_i_ (66.3%), NaHCO_3_-P_o_ (34.8%) and NaOH-P_i_ (59.0%) content, contributing to the transfer of P sources into NaHCO_3_-P_i_ and NaOH-P_i_ pools. This effect was achieved through elevated ALP (33.8%) and ACP (12.9%) activities and increased *pqqC* (48.1%), *phoC* (42.9%), *phoD* (21.7%), and *bpp* (27.4%) gene abundances. The Non-Leg treatment, on the other hand, led to significant increases in soil NaHCO_3_-P_i_ (299.0%) and NaHCO_3_-P_o_ (132.6%) content, thereby facilitating the turnover of P sources into NaHCO_3_-P_i_ and NaOH-P_i_ pools, except for the *phoC* gene abundance. Both Leg and Non-Leg treatments significantly improved citrus growth (7.3-20.0%) and P uptake (15.4-42.1%) in P-deficient soil but yielded no substantial effects in P-surplus soil. In summary, introducing green manure crops, particularly legume green manure, emerges as a valuable approach to enhance soil P availability and foster fruit tree growth in orchard production.

## 1 Introduction

The escalation of incomes and the expansion of the population over the past two decades are recognized as pivotal factors fostering the remarkable surge in global fruit consumption (Stratton et al., 2021). By the year 2021, the global expanse of orchards surpassed 64 million hectares, yielding a remarkable harvest of over 800 million tons (FAO, 2023). Phosphorus (P) holds the second position in terms of its significance among essential nutrient elements, following nitrogen (N), and assumes a critical function in the growth of fruit trees, as well as the yield and quality of fruits (Ahmed et al., 2019; Bibi et al., 2022). In order to uphold elevated plant yields and ensure global food security, approximately 19 million metric tons of phosphate rock-derived P are utilized annually for the production and application of fertilizers in agricultural systems (Chen and Graedel, 2016; Zhang et al., 2022). Nevertheless, the introduced P is readily bound by active metal ions present in soils, such as calcium (Ca^2+^), magnesium (Mg^2+^), iron (Fe^3+^), and aluminum (Al^3+^) cations, or adsorbed onto mineral surfaces. This leads to reduced P availability and diminished efficiency of P fertilizers (Fink et al., 2016b; Tian et al., 2020). The fixation and accumulation of P in soils pose a potential threat to ecological environments, including the occurrence of water eutrophication (Wang et al., 2019; Qin et al., 2020). In orchard production, these challenges are more pronounced due to the relatively low root length and density of fruit trees (typically around 2 cm·cm^-3^ for the root length to volume ratio) and their limited capacity to efficiently uptake soil nutrients (Kalcsits et al., 2020). Hence, enhancing the efficiency of P fertilizers and facilitating the conversion of accumulated P in the soil into bioavailable forms are crucial steps in promoting sustainable production and mitigating ecological risks in orchards.

Green manures, also referred to as cover crops, are generally grown at times when soil would otherwise be bare, typically in the period after a crop is harvested or the orchard alleyways (Lal, 2015). The utilization of green manure represents a significant field management strategy that can enhance the effectiveness of soil P and reduce the reliance on mineral P fertilizers (Li et al., 2015; Jamal et al., 2023; Ozbolat et al., 2023; Zhang et al., 2023). According to the study conducted by de Oliveira et al., 2017 in no-tillage onion production of Santa Catarina, Brazil, the incorporation of green manure residues has demonstrated the ability to promptly release soluble inorganic P (P_i_) and elevate the availability of P in the soil. The rate of P release was contingent upon the total P content and the C/N (Carbon to Nitrogen) ratio of the residues, as well as the activities of soil microorganisms involved in P solubilization. In a 6-year comprehensive trial of commercial soybean, maize, and wheat cultivation systems in southern Brazil, Soltangheisi et al. (2018) found that continuous tillage of a variety of green manures, including common vetch (*Vicia sativa*), white lupin (*Lupinus albus*), fodder radish (*Raphanus sativus*), ryegrass (*Lolium multiflorum*), and black oat (*Avena strigosa*) were effective in utilizing moderately labile P and increasing the proportion of labile P fractions in the soils. And, white lupin (*Lupinus albus*) exhibited the highest level of improvement. Similarly, Dube et al. (2014) found comparable outcomes in their research on the addition of grazing vetch (*Vicia dasycarpa L.*) and oats (*Avena sativa L.*) to maize-based conservation agriculture systems in the Eastern Cape Province of South Africa. Corroborating these findings, Gao et al. (2016) reported similar conclusions in their studies involving the utilization of alfalfa (*Medicago sativa L*) and broad bean (*Vicia faba L.*) in rice agroecosystems in eastern China. However, there is limited information available on the specific contribution of different green manure varieties, such as legume or non-legume species, to improving P availability in orchard ecosystems and the subsequent uptake by fruit trees.

Prior investigations have validated that the impact of incorporating green manure into the soil on the active P pool can be primarily attributed to two distinct factors. Firstly, there is the release of P from the green manure itself during the process of decomposition (Karasawa and Takahashi, 2015; Dong et al., 2021). Secondly, the addition of green manure as an external carbon (C) source can stimulate the growth and activity of P-cycling microorganisms (Sarker et al., 2019). The P-cycling microorganisms were considered to make a greater contribution to the soil biological P pool in the soil (Hallama et al., 2019), and they can facilitate the transformation and circulation of soil-insoluble P through processes such as solubilization of P_i_, mineralization of organic P (P_o_) and accumulation and turnover of biomass P (Kafle et al., 2019). But, the impact of soil microorganisms on soil P pools is regulated by the quality of the green manure (Fink et al., 2016a; Hansen et al., 2022). Previous studies have shown that green manure residues with high P concentrations (generally refer to legume green manure) decompose faster than residues with low P concentrations (generally refer to non-legume green manure) (Alamgir et al., 2012; Khan et al., 2022; Fontana et al., 2023). Asghar and Kataoka (2022) also indicated that the incorporation of soil with legume (*Vicia villosa*) green manure with a low C/P (Carbon to Phosphorus) ratio induced better soil P nutrition status and plant growth through increasing soil phosphatase and β-glucosidase activities and altering soil microbial community composition, compared to non-legume (*Brassica juncea L.*) green manure.

The impact of green manure on both the active P pools in the soil and the P nutrition of fruit trees is influenced not only by the C and P content of the green manure itself but also by the initial soil P status (Ullah et al., 2023). In the study by Maltais-Landry and Frossard (2015), the contribution of green manure residues to soil P fractions and a subsequent crop was similar and comparable to the effects of a water-soluble mineral P fertilizer, and with a greater contribution when soil initial available P was lower. Richardson and Simpson (2011) also indicated that under initial soil conditions of P deficiency, microorganisms have the ability to activate or deactivate various P-cycling genes and express microbial phosphatase enzymes. This activation leads to the mobilization of P pools that are not readily accessible, as opposed to initial soil conditions of P surplus. Nevertheless, there remains a lack of consensus regarding the influence of different green manure species on soil’s active P pools and the phosphorus nutrition of fruit trees, particularly in the context of differing initial soil P levels. Moreover, the mechanisms underlying these effects are not well understood.

In this study, a citrus pot experiment was conducted in soils characterized by both P surplus and deficiency. Green manure residues from both legume and non-legume sources were incorporated, and ^18^O-labeled KH_2_PO_4_ was introduced to trace the turnover characteristics of chemical P fertilizer facilitated by soil microorganisms. The objectives of this study were twofold: (1) to determine the magnitude of the effects of green manure additions on soil active P pools and citrus plant P uptake based on various green manure species and soil initial P status; (2) to unravel the microbiological mechanisms that underlie the aforementioned effects. Our hypothesis posited that the incorporation of green manure residues would markedly augment soil labile P pools and enhance P nutrition for citrus trees in both P-surplus and deficient soils. This effect was anticipated to occur through the stimulation of microorganisms and enzymes engaged in P cycling, leading to the conversion of accumulated soil P and chemical fertilizer P into forms readily available for crops. Additionally, we expected that the activation of legume green manures would surpass that of non-legume green manures in this context. This study can establish a theoretical foundation for the utilization of green manure in resenting a sustainable approach for the development of fruit tree production, contributing to long-term ecological balance and productivity.

## 2 Materials and methods

### 2.1 Experimental soil and crops

For the experiment, soils with both P-surplus and P-deficient were selected. These soils were obtained from Danling County (30°04′ N, 103°53′ E), in Sichuan Province of China. The P-surplus soil was collected from a well-established citrus orchard, while the P-deficient soil was obtained from a recently established citrus orchard. Based on the classification by the United States Department of Agriculture Soil Taxonomy, both the experimental soils were classified as Alfisols. Soils used in the containers were taken from the top 20 cm of soil layer in October 2020. They were sieved to < 2 mm after air-drying and removing visible plant residues and stones. The fundamental characteristics of the soil samples were presented in **Table 1**.

**Table 1:** The properties of P-surplus and P-deficient soils used in this experiment.

Soil properties	P-surplus soil	P-deficient soil
TP (g·kg^-1^)	1.50 ± 0.09 a	0.17 ± 0.01 b
AP (mg·kg^-1^)	274.40 ± 16.02 a	4.20 ± 0.15 b
TN (g·kg^-1^)	2.39 ± 0.09 a	0.81 ± 0.05 b
TK (g·kg^-1^)	15.28 ± 0.24 a	15.93 ± 0.41 a
SOM (g·kg^-1^)	37.40 ± 2.07 a	18.98 ± 1.66 b
pH	5.40 ± 0.19 a	4.10 ± 0.06 b

TP, soil total phosphorus; AP, soil available phosphorus; TN, soil total nitrogen; TK, soil total potassium; SOM, soil organic matter; pH, measured in 1:2.5 soil/water suspensions. The soils of this experiment were categorized according the soil nutrient classification standard for citrus orchards, with AP < 5.0 mg/kg as extremely deficient, AP 5.0 – 15.0 mg/kg as deficient, AP 15.0 – 80.0 mg/kg as moderate, and AP > 80.0 mg/kg as excessive. Values in the same row within the same parameters followed by diﬀerent letters were signiﬁcantly diﬀerent at *p* < 0.05 according to Tukey’s test.

The citrus employed in the present study was of the first-year seedling stage, and the species is the Ehime mandarin 38^th^. The legume and non-legume green manure residues were hairy vetch (*Vicia villosa*) and rattail fescue (*Vulpia myuros (L.) C.C. Gmel.*), respectively. The green manures were freshly harvested from the field in March, 2021 and then finely chopped into 2 mm in size. The nutrient content of the green manure residues was presented in **Table 2**.

**Table 2:** The nutrient content of the green manure residues (%, calculated by dry mass).

Green manure types	Carbon	Nitrogen	Phosphorus	Potassium	C/N ratio	C/P ratio
Leg	43.26 ± 2.83 a	3.95 ± 2.03 a	0.59 ± 0.09 a	7.09 ± 0.11 a	10.95 ± 1.24 b	73.32 ± 2.13 b
Non-Leg	43.61 ± 0.49 a	1.86 ± 0.25 b	0.31 ± 0.13 b	3.83 ± 0.06 b	23.49 ± 0.53 a	140.67 ± 4.33 a

Leg, legume green manure [hairy vetch (*Vicia villosa*)]; Non-Leg, non-legume green manure [rattail fescue (*Vulpia myuros* (L.) C.C. Gmel.)]. C/N ratio, Carbon to Nitrogen ratio in green manure residues. C/P ratio, Carbon to Phosphorus ratio in green manure residues. Values in the same column within the same parameters followed by diﬀerent letters were signiﬁcantly diﬀerent at *p* < 0.05 according to Tukey’s test.

### 2.2 Experimental design and sampling

A pot-based experiment was conducted from March to September 2021 for the present study, which was carried out within a greenhouse facility located at Southwest University (29°81′ N, 106°42′ E), Chongqing, China. The experiment was designed using a completely randomized approach, considering two primary factors: (1) two treatments related to soil P levels, specifically P-surplus and P-deficient soils; (2) three treatments related to addition materials, including legume green manure (Leg), non-legume green manure (Non-Leg) and a control treatment with no addition (CK). A fixed quantity of 2 g·C·kg^-1^ (dry soil) of green manure was added in all treatments. Due to the significant individual variations and potential systematic errors in fruit tree cultivation, each treatment was replicated five times. Each pot was filled with 10 kg of mixed soils, green manure residues and chemical fertilizers (40 mg·kg^-1^ N, 40 mg·kg^-1^ P_2_O_5_ and 80 mg kg^-1^ K_2_O were used as the foundation fertilizers, 40 mg·kg^-1^ N were used as top-dress to ensure sufficient nutrition to citrus growth). The N, P and K fertilizers used in this study was urea, KH_2_PO_4_ and K_2_SO_4_, respectively. In addition, 0.5 g KH_2_P^18^O_4_ (containing 80‰ δ^18^O_p_, diluted from KH_2_P^18^O_4_ (^18^O_4_, 95%), purchased from Cambridge Isotope Laboratories) were applied to each pot to track the fate of exogenous P fertilizer. Ehime mandarin 38^th^ citrus seedlings were subsequently transplanted into pots and watered daily to 60% of the field water capacity. The position of pots switched once a week to minimize possible environmental effects.

Destructive sampling was conducted in September 2021, after citrus summer tips turned green. Plant samples were collected by separating the roots, stems, new leaves, and old leaves, following measurements of citrus height, stem thickness, and biomass. The collected plant samples were subjected to oven-drying at 105°C for 30 minutes, followed by further oven-drying at 70°C until a constant weight was achieved. Subsequently, the dried samples were ground and sieved through a 0.5 mm mesh size to determine the plant P concentration. The soil samples were divided into two portions. One portion was air-dried for the determination of total P and available P content, while the other portion was preserved at -80°C. The preserved samples were later used for analyzing phosphatase activity, the abundance of P-cycling genes, and the ^18^O_P_ values of different forms of P_i_.

### 2.3 Determination of P in soil and plant samples

Soil total P and available P content were determined by molybdenum antimony anti-colorimetric method after digested with NaOH and extracted by NH_4_F-HCl, respectively (Murphy and Riley, 1986). Different soil P fractions were sequentially extracted following a modified Hedley method (Hedley et al., 1982). Briefly, 0.5 g dried soil was sequentially extracted with 30.0 mL Milli-Q water (most labile P), 0.5 mol·L^-1^ NaHCO_3_ (pH 8.5) (labile and weakly adsorbed P), 0.1 mol·L^-1^ NaOH (Fe/Al oxide-bound P) and 1.0 mol·L^-1^ HCl (Ca-P minerals) after shaking overnight (16 h) at 25°C at 165 rpm. The supernatants were collected by centrifugation (10 min at 5000 rpm) and then filtered through 0.45 mm cellulose-acetate filters membrane to determine total P (P_t_) and P_i_ content, the difference between P_t_ and P_i_ was taken as P_o_ content. Residual-P was determined by the molybdenum-antimony colorimetry after digested using the mixture of H_2_O_2_ and H_2_SO_4_.

Plant P concentration was measured by the molybdovanado phosphate method after digested in concentrated H_2_SO_4_ and H_2_O_2_ (Thomas et al., 1967).

### 2.4 Measurement of soil enzyme activity

Soil acid phosphatase (ACP) and alkaline phosphatase (ALP) activities were determined by the disodium phenyl phosphate colorimetric method (Jin et al., 2016). The ACP was extracted using acetate buffer at pH 5.0, while the ALP was extracted using borate buffer at pH 10.0.

### 2.5 Extraction of soil DNA and quantification of P-cycling genes

The fresh soil samples (0.5 g) was subjected to whole genomic DNA extraction using FastDNA ^®^ Spin Kit (MP Biomedical, Santa Ana, USA). DNA purity and quality were detected by NanoDrop ND-2000 spectrophotometer and nucleic acid integrity was determined by agarose gel electrophoresis. The tested P-cycling genes, including *pqqC* (pyrroloquinoline-quinone synthase), *phoD* (alkaline phosphatase D), *phoC* (acid phosphatase C) and *bpp* (β-helical phytase), as well as 16S rRNA gene as a reference for bacterial abundance were quantified by quantitative PCR (qPCR) reations via QuantStudioTM 6 Flex Real-Time System (Applied Biosystems, USA). All primers used in this study were listed in **Table 3**. The amplification program initialized with 95°C for 10 min, following with 40 cycles of 95°C for 5 s, 58°C for 30 s and 72°C 1 min. Each PCR assay was performed in triplicate, with amplification efficiencies between 91-100%. The copy number of each target gene in soil DNA was calculated based on the standard curve and the mean Ct value. The relative abundance of P-cycling genes was calculated as the ratio of cope numbers of respective P-cycling gene and 16S rRNA gene.

**Table 3:** The primers used for quantitative qPCR and corresponding amplification cycling conditions in this experiment.

Primer set	Target gene	Amplicon length (bp)	Amplification efficiencies	Amplification cycling conditions	References
pqqC-F (CATGGCATCGAGCATGCTCC)	*pqqC*	546	94.06%	40 cycle (95°C 5 s, 58°C 30 s, 72°C 1 min)	(Meyer et al., 2011)
pqqC-R (CAGGGCTGGGTCGCCAACC)					
ALPS-F730 (CAGTGGGACGACCACGAGGT)	*phoD*	371	99.87%		(Luo et al., 2020; Sakurai et al., 2008)
ALPS-R1101 (GAGGCCGATCGGCATGTCG)					
phoC-A-F1 (CGGCTCCTATCCGTCCGG)	*phoC*	155	97.55%		(Fraser et al., 2017; Gaiero et al., 2018)
phoC-A-R1 (CAACATCGCTTTGCCAGTG)					
BPP-F (GACGCAGCCGAYGAYCCNGCNITNTGG)	*bpp*	175	91.71%		(Huang et al., 2009)
BPP-R (5′-CAGGSCGCANRTCIACRTTRTT-3′)					
338F (GGGTTGCGCTCGTTGC)	16S rRNA	191	96.95%		(Zhang et al., 2019)
518R (ATGGYTGTCGTCAGCTCGTG)					

### 2.6 Measurement of oxygen isotope ratios in phosphate

The measurement of oxygen isotope ratios in phosphate was followed the methods of Bi et al. (2018) and Jiang et al. (2017). In brief, 25.0 g freeze-dried fresh soil was sequentially extracted with H_2_O, NaHCO_3_, NaOH and HCl. Frist, the magnesium-induced co-precipitation (MAGIC) method was used to enrich PO_4_^3-^ and added DAX-8 macroporous resin to eliminate the organics in samples. Then, the coprecipitation of ammonium phosphomolybdate (APM) and magnesium ammonium phosphate (MAP) method was used to further separated and purified PO_4_^3-^. After anion cation resin purified, PO_4_^3-^ was converted into Ag_3_PO_4_ precipitation by ammonia volatilization method. Finally, the mineral structure of Ag_3_PO_4_ was detected by X-ray diffractometer (XRD) and compared with the standard pattern to check the purity of the sample and the soil ^18^O_P_ values were measured after the sample passing the test.

### 2.7 Statistical analysis

The values presented in the figures and tables are given as means ± standard errors. One-way analysis of variance (ANOVA) tests was used to examine the significant changes in soil P fractions, enzyme activities and the microbial gene abundance, citrus growth parameters and citrus P uptake under CK, Leg and Non-Leg treatments in P-surplus and P-deficient soils, respectively. Spearman correlation analysis was conducted to reveal the relationship between soil P fractions, the δ^18^O_P_ values of P_i_ pools and phosphatase activities, P-cycling gene relative abundance in P-surplus and P-deficient soils, respectively. All the statistics analysis were performed with IBM SPSS Statistics 22.0.

To evaluate the direct and indirect factors (including green manure addition, soil phosphatase activities and P-cycling genes abundances) affecting soil P sources transformation and citrus P uptake under different treatments in P-surplus and P-deficient soils, partial least squares path models (PLS-PM) were constructed using the R 4.2.3 packages “vegan” and “plspm”. And all figures in the present study were plotted using GraphPad Prism 8.0 and Adobe Illustrator 2023.

## 3 Results

### 3.1 Citrus growth and P uptake

**Figure 1:**
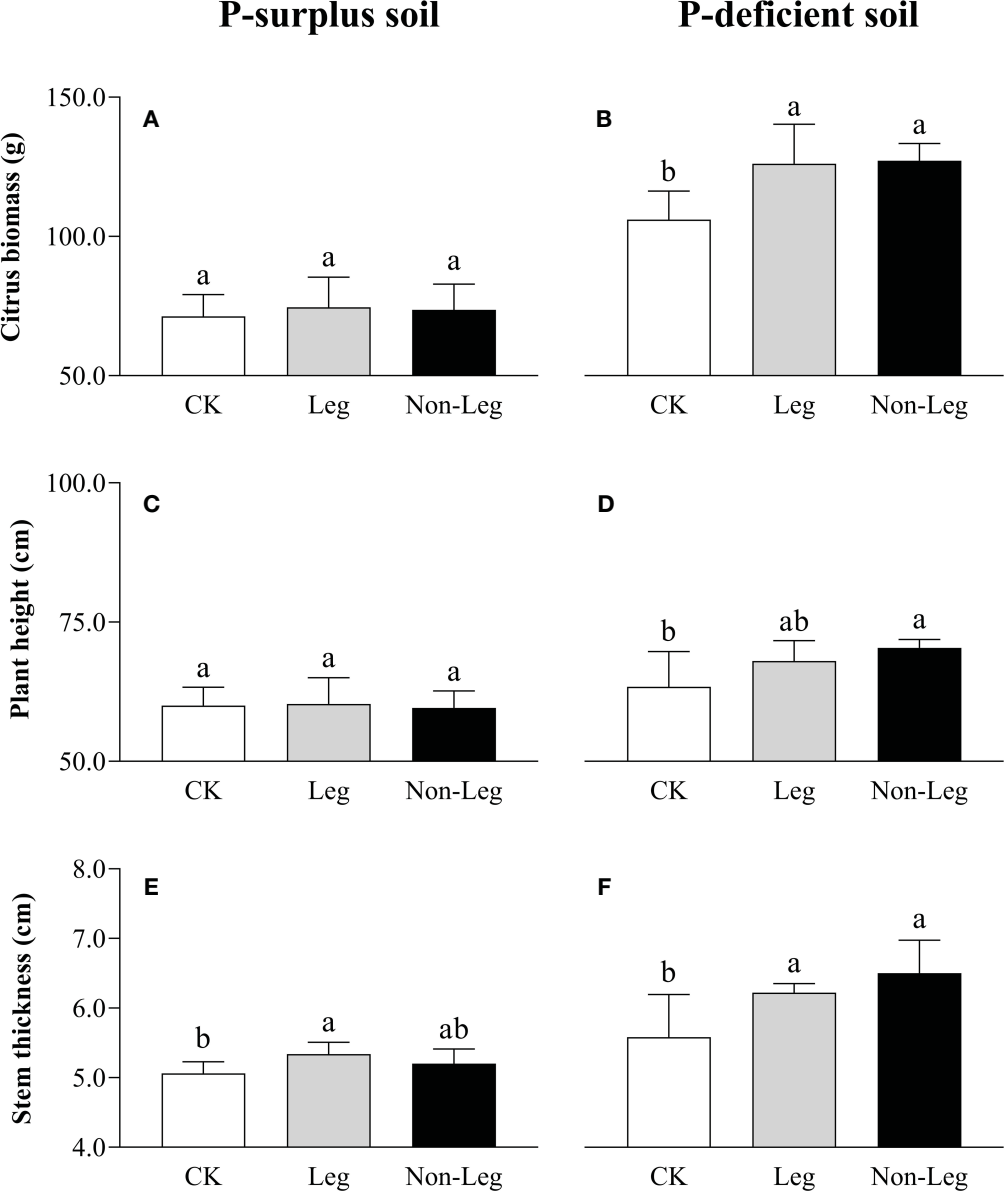
The citrus biomass **(A, B)**, plant height **(C, D)** and stem thickness **(E, F)** under different treatments in P-surplus and P-deficient soils. CK, no green manure residues addition; Leg, legume green manure residues addition; Non-Leg, non-legume green manure residues addition. Vertical bars indicated the standard errors. Different letters indicated significant differences among treatments at *p* < 0.05.

In comparison to the control treatment (CK), the addition of green manure residues did not yield a statistically significant impact on the growth of citrus plants in P-surplus soil, except for a 5.5% increase (*p* < 0.05) in stem thickness observed under the Leg treatment (**Figure 1E**). However, both citrus biomass and stem thickness exhibited significant increases (*p* < 0.05) under both Leg (19.0% and 11.5%, respectively) and Non-Leg (20.0% and 16.5%, respectively) treatments in P-deficient soil (**Figures 1B, F**). Additionally, plant height also experienced a significant increase (*p* < 0.05) under the Non-Leg treatment, with an 11.1% increase (**Figure 1D**).

**Figure 2:**
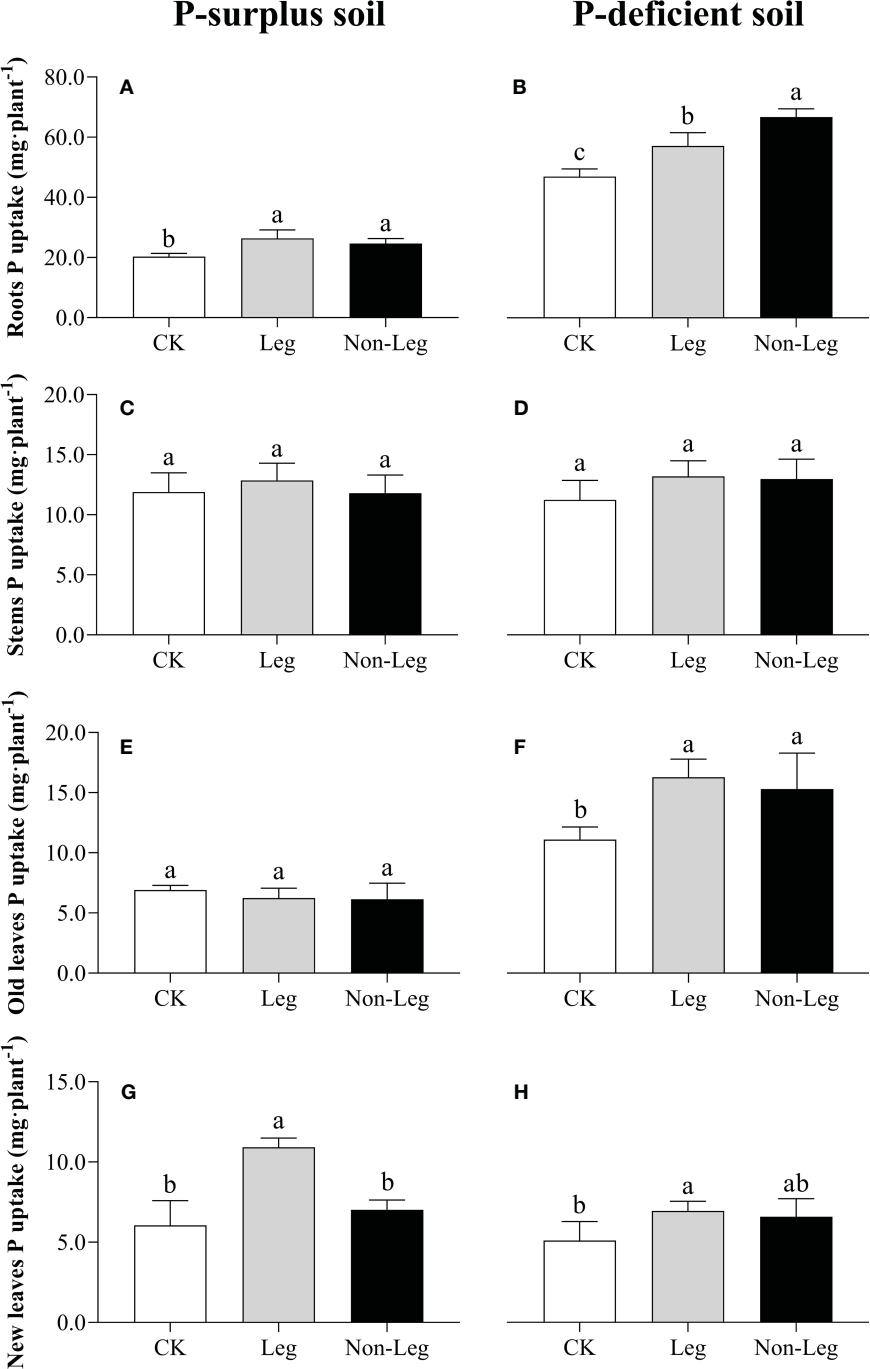
Phosphorus uptake in citrus roots **(A, B)**, stems **(C, D)**, old leaves **(E, F)** and new leaves **(G, H)** under different treatments in P-surplus and P-deficient soils. CK, no green manure residues addition; Leg, legume green manure residues addition; Non-Leg, non-legume green manure residues addition. Vertical bars indicated the standard errors. Different letters indicated significant differences among treatments at *p* < 0.05.

In P-surplus soil, the addition of legume green manure residues significantly increased P uptake of citrus roots and new leaves by 29.6% and 80.1% (*p* < 0.05), respectively (**Figures 2A, G**). And, a 21.3% increase (*p* < 0.05) of P uptake in citrus roots by Non-Leg treatment was also observed (**Figure 2A**). In P-deficient soil, there were significant increases (*p* < 0.05) in P uptake observed in citrus roots and old leaves under both the Leg and Non-Leg treatments, compared to the control treatment (CK). Specifically, there was a 21.7% increase in citrus roots and a 46.7% increase in old leaves under the Leg treatment, while the Non-Leg treatment showed a 42.1% increase in citrus roots and a 37.9% increase in old leaves (**Figures 2B, F**). Additionally, P uptake in new leaves significant increased (*p* < 0.05) 36.3% by Leg treatment (**Figure 2H**).

### 3.2 Soil different P fractions

In P-surplus soil, the H_2_O-P_i_ and NaHCO_3_-P_o_ content demonstrated a significant increase (*p* < 0.05) under both Leg (13.6% and 8.9%, respectively) and Non-Leg (9.2% and 8.5%, respectively) treatments. Furthermore, the NaOH-P_i_ and NaOH-P_o_ content also significantly increased by 9.5% and 30.0% (*p* < 0.05) under the Leg treatment, respectively (**Table 4**).

**Table 4:** The content of soil P fractions under different treatments in P-surplus and P-deficient soils.

	P fractions (mg kg^-1^)	CK	Leg	Non-Leg
P-surplus soil	H_2_O-P_i_	117.78 ± 7.22 b	133.83 ± 2.84 a	128.23 ± 6.05 a
	NaHCO_3_-P_i_	370.35 ± 16.50 a	381.08 ± 3.72a	381.45 ± 6.88 a
	NaOH-P_i_	565.46 ± 58.97 b	619.35 ± 14.91 a	591.40 ± 3.66 ab
	HCl-P_i_	1109.28 ± 18.73 a	1086.88 ± 29.64 a	1088.63 ± 45.51 a
	NaHCO_3_-P_o_	306.70 ± 21.82 b	335.00 ± 32.99 a	332.86 ± 20.82 a
	NaOH-P_o_	97.79 ± 11.08 b	126.88 ± 16.60 a	116.50 ± 6.77 ab
	Residual-P	482.36 ± 34.02 a	491.57 ± 25.96 a	490.60 ± 34.40 a
P-deficient soil	H_2_O-P_i_	0.06 ± 0.02 b	0.15 ± 0.04 a	0.14 ± 0.09 ab
	NaHCO_3_-P_i_	4.42 ± 0.36 c	7.35 ± 0.78 a	5.96 ± 1.03 b
	NaOH-P_i_	23.09 ± 2.76 b	36.71 ± 4.47 a	24.79 ± 5.48 b
	HCl-P_i_	0.87 ± 0.21 a	1.04 ± 1.04 a	0.97 ± 0.25 a
	NaHCO_3_-P_o_	0.95 ± 0.95 c	3.79 ± 0.92 a	2.21 ± 0.56 b
	NaOH-P_o_	36.83 ± 2.35 a	37.98 ± 2.40 a	37.37 ± 3.63 a
	Residual-P	111.94 ± 7.32 a	113.74 ± 5.25 a	115.83 ± 4.97 a

P_i_, inorganic phosphorus; P_o_, organic phosphorus. CK, no green manure residues addition; Leg, legume green manure residues addition; Non-Leg, non-legume green manure residues addition. Values in the same row within the same parameters followed by diﬀerent letters were signiﬁcantly diﬀerent at *p* < 0.05 according to Tukey’s test.

In P-deficient soil, the NaHCO_3_-P_i_ and NaHCO_3_-P_o_ content showed a significant increase (*p* < 0.05) under both Leg (66.3% and 34.8%, respectively) and Non-Leg (299.0% and 132.6%, respectively) treatments. Additionally, the H_2_O-P_i_ and NaOH-P_i_ content also significantly increased by 150.0% and 59.0% (*p* < 0.05) under the Leg treatment, respectively (**Table 4**).

The content of HCl-P_i_ and Residual-P did not significantly change among the different treatments in both P-surplus and P-deficient soils.

### 3.3 The oxygen isotope ratios in different soil P_i_ pools

**Figure 3:**
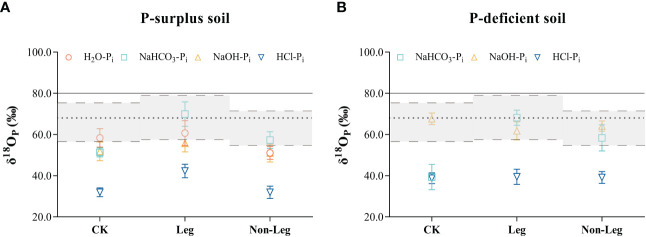
The oxygen isotope ratios (δ^18^O_P_) in different soil inorganic P (P_i_) pools among different treatments of both P-surplus **(A)** and P-deficient **(B)** soils. CK, no green manure residues addition; Leg, legume green manure residues addition; Non-Leg, non-legume green manure residues addition. The grey zones with long-dotted line represented equilibrium range calculated based on the monthly average highest and lowest soil temperatures and soil water isotopes values (δ^18^O_w_) under different treatments during the pot experiment. The short-dotted line was the average equilibrium value calculated using the average soil temperature and average δ^18^O_w_, which was included in the equilibrium zones. The δ^18^O_P_ value of superphosphate was indicated with solid line. Vertical bars indicated the standard errors. The δ^18^O_P_ values of H_2_O were not detected in P-deficient soil in the present study.

Green manure residues addition changed the phosphate oxygen isotope ratios of different P_i_ pools in both P-surplus and P-deficient soils (**Figure 3**). In P-surplus soil, compared with CK, the δ^18^O_P_ values of NaHCO_3_-P_i_ and NaOH-P_i_ under the Leg treatment, as well as NaHCO_3_-P_i_ under the Non-Leg treatment, tended to approach or shift toward the isotopic equilibrium zone (**Figure 3A**). This indicated that the biological cycling of these P_i_ pools may be rapid and related to the oxygen isotope equilibration. In addition, the δ^18^O_P_ values of HCl-P_i_ under the Leg treatment would be lighter than the other treatments.

In P-deficient soil, the phosphate oxygen isotope ratios of various P_i_ pools exhibited similar variations under both Leg and Non-Leg treatments. Compared to CK, the δ^18^O_P_ values of NaHCO_3_-P_i_ showed a considerable increase (shifted toward the isotopic equilibrium zone) following the addition of green manure residues (**Figure 3B**). This observation suggests that microorganisms play a crucial role in the turnover of NaHCO_3_ pools.

### 3.4 Soil microbial activities

**Figure 4:**
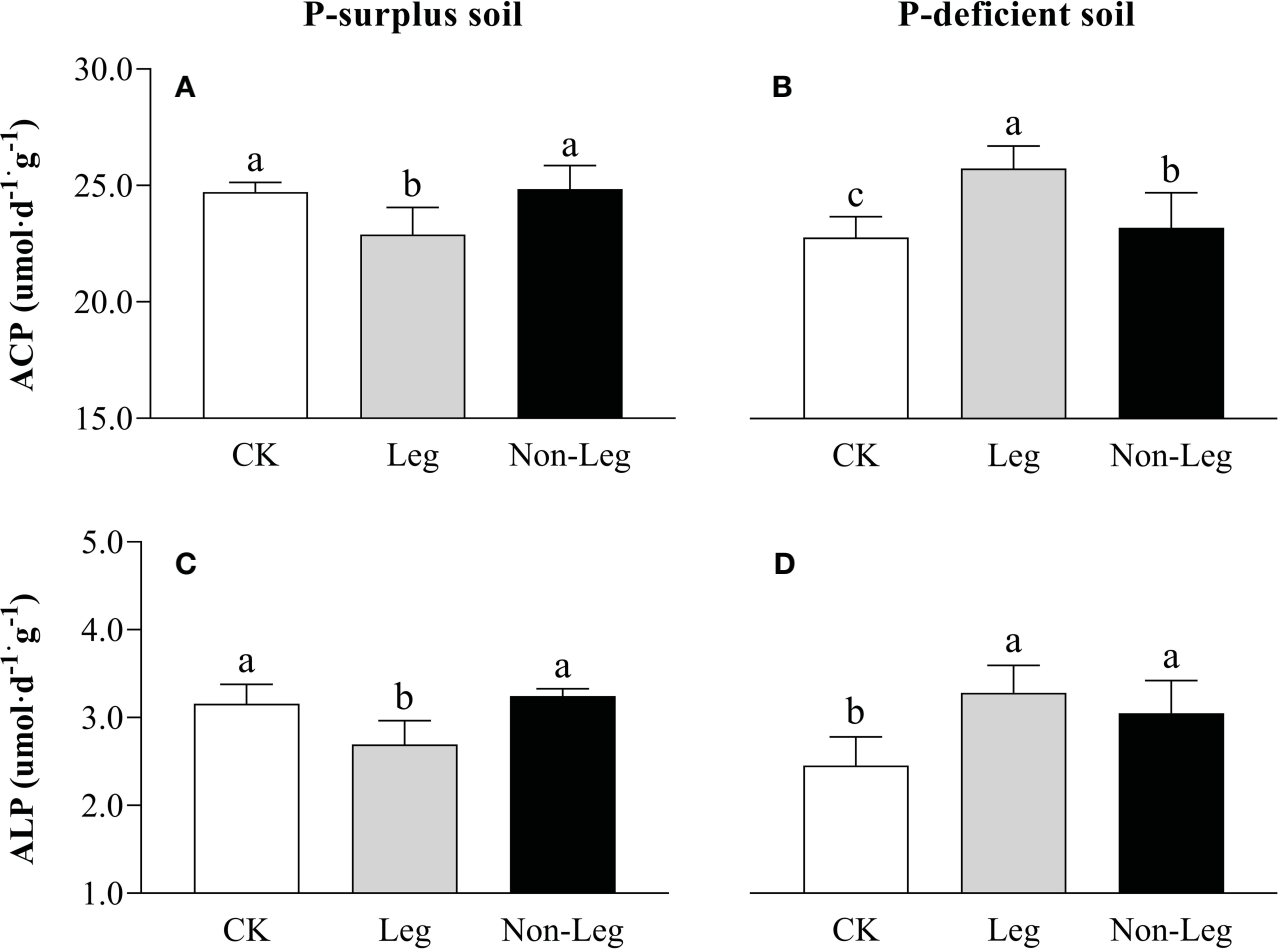
The ACP **(A, B)** and ALP **(C, D)** activities under different treatments in P-surplus and P-deficient soils. CK, no green manure residues addition; Leg, legume green manure residues addition; Non-Leg, non-legume green manure residues addition. Vertical bars indicated the standard errors. Different letters indicated significant differences among treatments at *p* < 0.05.

In P-surplus soil, the activities of ACP and ALP exhibited a significant decrease (*p* < 0.05) under the Leg than CK treatment, while no significant change was observed by treated with Non-Leg (**Figures 4A, C**). Conversely, in P-deficient soil, the activities of soil ACP and ALP significantly increased (*p* < 0.05) under both Leg and Non-Leg than CK treatments, with a higher increase observed under the Leg treatment (**Figures 4B, D**).

**Figure 5:**
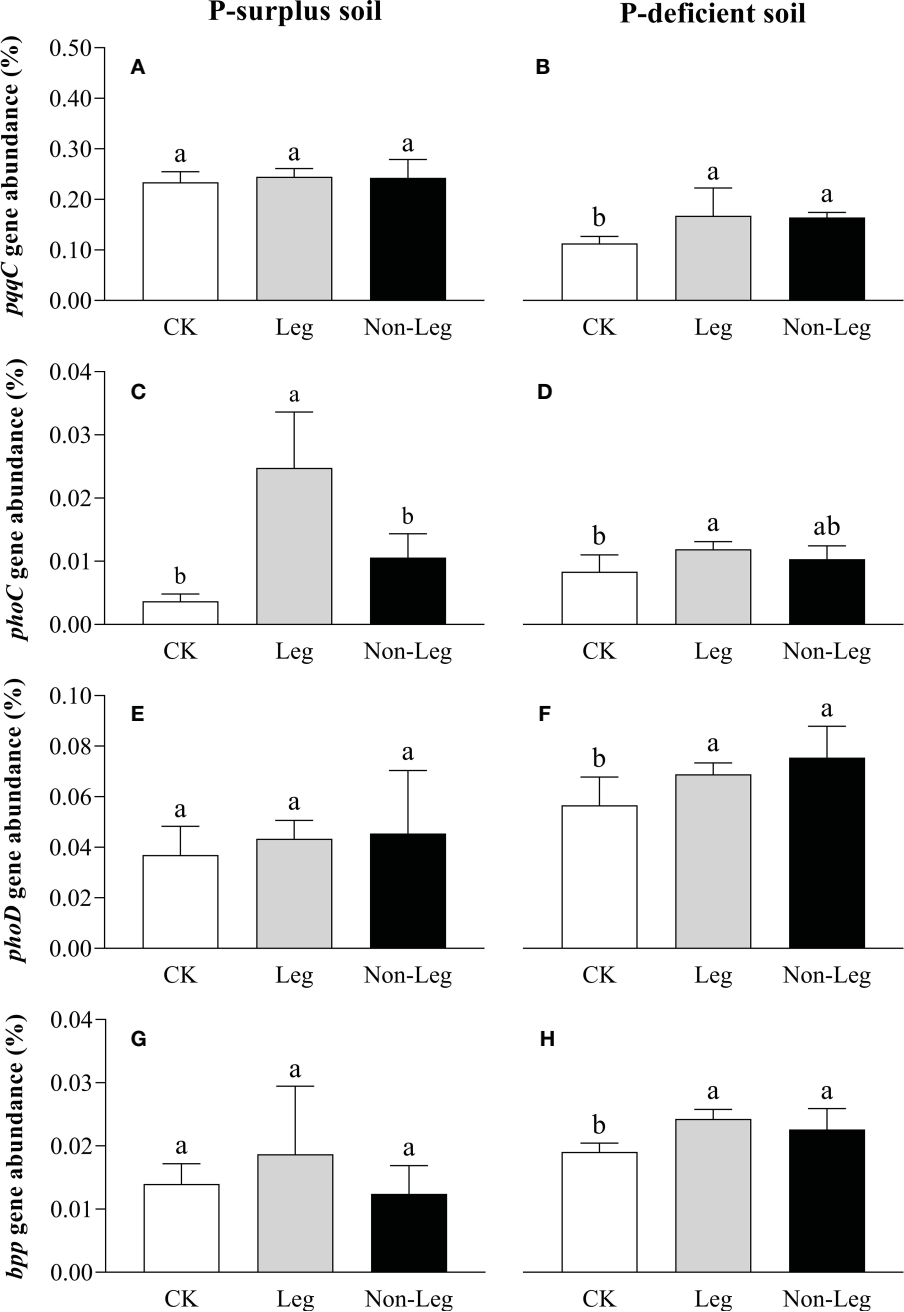
The relative abundance of *pqqc* **(A, B)**, *phoC* **(C, D)**, *phoD* **(E, F)** and *bpp* **(G, H)** under different treatments in P-surplus and P-deficient soils. The relative abundance of P-cycling genes was calculated as the ratio of cope numbers of respective P-cycling gene and 16S rRNA gene. CK, no green manure residues addition; Leg, legume green manure residues addition; Non-Leg, non-legume green manure residues addition. Vertical bars indicated the standard errors. Different letters indicated significant differences among treatments at *p* < 0.05.

In P-surplus soil, the addition of green manure residues did not have a significant effect on the relative abundances of soil P-cycling genes, except for the *phoC* gene. Specifically, under the Leg treatment, the relative abundance of the *phoC* gene exhibited a significant increase (*p* < 0.05) of 576.6% compared to CK (**Figures 5A, C, E, G**). In P-deficient soil, compared to CK, the relative abundance of *pqqC*, *phoC*, *phoD* and *bpp* genes under the Leg treatment significantly increased (*p* < 0.05) by 48.1%, 42.9%, 21.7% and 27.4%, respectively. And the relative abundance of *pqqC*, *phoD* and *bpp* genes under the Non-Leg treatment significantly increased (*p* < 0.05) by 45.1%, 33.3% and 18.6%, respectively (**Figures 5B, D, F, H**).

### 3.5 Relative contributions of various factors to soil P fractions and citrus P uptake

**Figure 6:**
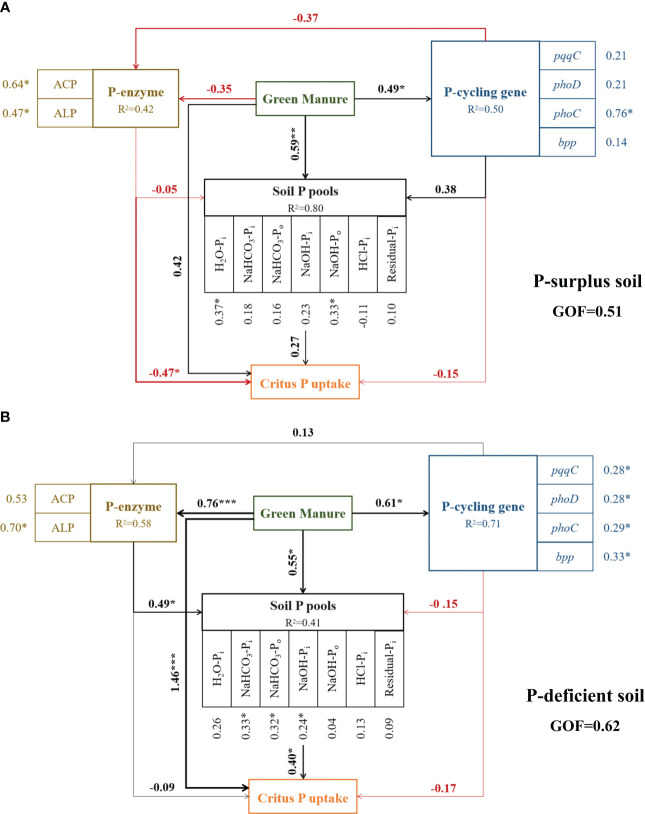
Evaluation of factors affecting soil P fractions and citrus P uptake under different treatments in P-surplus **(A)** and P-deficient **(B)** soils by partial least squares path modeling (PLS-PM). R^2^ denotes the proportion of variance explained. The black and red lines indicate positive and negative relationships, respectively. The width of the arrows is proportional to the strength of the path coefficients. * indicated significant at *p* < 0.05, ** indicated significant at *p* < 0.01, *** indicated significant at *p* < 0.001.

In P-surplus and P-deficient soils, the effects of green manure addition on soil P fractions and citrus P uptake were correlated with the majority microbial parameters in P-cycling, including enzymes activities and P-cycling genes abundances (**Figure 6**).

In P-surplus soil, the addition of green manure residues had a positive impact on the turnover of soil P fractions through direct effects (path coefficient = 0.59). Additionally, the influence of green manure residues on citrus P uptake was mediated indirectly through alterations in soil phosphatase activities, including both ACP and ALP (**Figure 6A**).

In P-deficient soil, the addition of green manure residues had a positive influence on the turnover of soil P fractions through both direct effects (path coefficient = 0.55) and indirect effects, mediated by alterations in soil phosphatase activities (path coefficient = 0.49). These changes in soil phosphatase activities or soil P pools, in turn, affected citrus P uptake (path coefficient = 0.40). Additionally, citrus P uptake was also directly influenced by the addition of green manure residues (path coefficient = 1.46) (**Figure 6B**).

## 4 Discussion

### 4.1 The changing of soil P pools

This study confirmed green manure residues incorporation had a positive effect on the enhancement of soil P pools (**Figure 6**; **Table 4**). This effect was mediated by both green manure species and initial soil P levels, characterized that legume green manure had a superior capacity to regulate soil P pools compared to non-legume green manure, and was more prominent in P-deficient soil.

Green manure residues contain considerable amounts of nutrients and their decomposition results in the release of these nutrients into the soil, which can assist in fulfilling the nutritional requirements of crops (Calegari et al., 2013; Jamal et al., 2023). Hinsinger et al. (2011) and Pavinato et al. (2008) indicated that organic forms of P released during residues decomposition were less prone to strong adsorption on the functional groups of Fe and Al oxides and hydroxides compared to inorganic forms. And organic decomposition products can also compete for adsorption sites, thereby increasing bioavailable of soil P pool (Varela et al., 2017; Soltangheisi et al., 2020). Maltais-Landry and Frossard (2015) also reported that comparable transfer of P from green manure residues and water-soluble mineral fertilizer to the soil was observed. Consistently, Hallama et al. (2019) confirms the observation of this study, that legume green manure was found to be more nutrient efficient than non-legume green manure. This can be primarily attributed to the following reasons. Firstly, legume green manure exhibits a higher capacity for P uptake and accumulation due to its abundant biomass (Arrobas et al., 2015). In regard to the green manure materials supplied by this study, when subjected to equal carbon input (2 g·C·kg^-1^ soil), the P input of legume green manure was 270 mg·P·pot^-1^, while the P input of non-legume green manure was 140 mg·P·pot^-1^ (**Table 2**). Consequently, there is a greater release of P during decomposition, thereby replenishing the labile soil P pools and promoting the growth of the main plant. Secondly, legume green manure with lower C/N and C/P ratios can more rapidly facilitate microbial decomposition and utilization, as well as stimulate the activity of P-cycling microorganisms (Truong and Marschner, 2020). This leads to the formation of greater microbial biomass P (Benitez et al., 2016), which is considered a potential active P pool in the soil (Hallama et al., 2019). These two factors collectively increased soil active P fraction (e.g. H_2_O-P_i_, NaHCO_3_-P_o_, NaOH-P_i_ and NaOH-P_o_ in P-surplus soil and H_2_O-P_i_, NaHCO_3_-P_i_, NaHCO_3_-P_o_ and NaOH-P_i_ in P-deficient soil (**Table 4**) and enhanced the growth and P nutrition of citrus plants (**Figures 1**, **2**).

The impact of green manure on soil P fractions were greatly influenced by the soil condition, particularly the size of the easily and sparingly available P pool (Damon et al., 2014). Similar to the finding of this study, the meta-analysis conducted by Hallama et al. (2019) reported that the benefits of green manure in terms of soil biological P were more pronounced in soils that had limited availability of P compared to sites with higher P availability. This study provided evidence that the contribution of green manure residues to labile soil P pools and citrus growth was comparatively limited in the context of the already abundant P availability in the soil (**Figures 1**, **2**, **6**; **Table 4**). This perspective was also confirmed by the research of Rick et al. (2011). Their study demonstrated that incorporation of green manure and input of rock P fertilizer had no significant impact on labile soil P fractions, wheat biomass, or P concentration due to high soil initial P availability, which already satisfied the requirements for crop growth.

### 4.2 The changing of soil P-cycling microorganism

There is a consensus that the incorporation of green manure residues into soil can serve as a source of C and energy for soil microorganisms (Huang et al., 2021), which in turn promotes increased metabolic activities of P-cycling microbial populations and facilitates the conversion of bio-unavailable P into bio-available forms (Pavinato et al., 2017; Zhou et al., 2018; Arruda et al., 2021). In the current study, the effects of microorganisms on soil P cycling were investigated using oxygen isotope labeling on exogenous chemical P fertilizers. This labeling technique allowed for tracking and monitoring the behavior and transformations of P_i_ within soil, and the closer isotope value of a P pool was to the theoretical equilibrium, the faster that the P pool was biologically cycled, indicating higher bioavailability. Conversely, if the isotope value deviated from the equilibrium, it suggested slower biological cycling and lower bioavailability of P (Bi et al., 2018; Tian et al., 2020). This study proved that incorporating green manure increased the conversion of chemical P fertilizer by microorganisms. In P-surplus soil, microorganisms quickly transformed P sources into H_2_O-P_i_ and NaHCO_3_-P_i_ pools under the Leg treatment and into NaHCO_3_-P_i_ pools under the Non-Leg treatment (**Figure 3A**). In P-deficient soil, both Leg and Non-Leg treatments promoted the conversion of P sources into NaHCO_3_-P_i_ and NaOH-P_i_ pools (**Figure 3B**). These findings provide direct validation of the pivotal role played by green manure in the mobilization of soil P through the activation of soil microorganisms. This activation is achieved by increasing the abundance of P-cycling bacteria and enhancing the activities of P-cycle enzymes (Piotrowska-Dlugosz and Wilczewski, 2020; Hallama et al., 2021). By promoting these microbial processes, green manure contributes to the efficient cycling and availability of phosphorus in the soil ecosystem.

In the present study, green manure residues addition had a positive effect on *pqqC*, *phoC*, *phoD* and *bpp* genes abundances and ACP and ALP activities, which was more prominent in P-deficient soil (**Figures 4**, **5**). In response to a P_i_-limited soil environment, microorganisms tend to release a higher quantity of phosphatases (Bergkemper et al., 2016). These enzymes are capable of effectively hydrolyzing ester-phosphate bonds in various phosphate-esters, thereby promoting the mineralization of soil insoluble P. This process enhances the overall effectiveness of P in the soil and facilitates its availability for biological uptake and cycling (Lu et al., 2022; Touhami et al., 2023).

Moro et al. (2021) also observed that in the soil with low P_i_ availability, microorganisms would be stimulated, especially bacteria, to synthesize large amounts of phosphatases, including acid phosphomonoesterase and phosphodiesterase, which promoted the hydrolysis of soil P_o_, replenished soil active P pools and supplied plant growth. In contrast to the expected response, the incorporation of legume green manure in P-surplus soil, as observed in this study, resulted in a decrease in ACP and ALP activities (**Figures 4A, C**). Indeed, the study by Spohn and Kuzyakov (2013) indicated that in soils with ample P availability, soil microbial biomass P was primarily influenced by the release of plant-available P as a by-product of C mineralization. The incorporation of legume green manure in P-surplus conditions lead to an increased availability of plant-available P, reducing the need for microbial phosphatase activity (**Table 4**). This finding could potentially explain the observed decrease in phosphatase activities under the Leg treatments in P-surplus soil of present study (**Figures 4A, C**).

The results of this study, except for the *phoC* gene in P-surplus soil, showed no significant differences in the abundance of other P-cycling genes between the incorporation of legume and non-legume green manure treatments (**Figure 5**). This finding contradicts our understanding that organic matter with a low C/P ratio, typically found in legume green manure, has a greater capacity to stimulate P-microbial abundance compared to organic matter with a high C/P ratio, typically found in non-legume green manure (Benitez et al., 2016; Erinle and Marschner, 2020; Erinle et al., 2020). It is worth noting that the C/P ratio of the legume green manure and non-legume green manure used in this study was 73 and 141, respectively, both below 200 (**Table 2**). These ratios fall within the same range, which could explain the limited differences observed in P-cycling genes abundances. Alamgir et al. (2012) pointed out that crop residues with a C/P ratio above 200 tend to induce net P immobilization and depletion of P pools, while residues with a C/P ratio below 200 increase P availability and P pools. Additionally, Blanco-Canqui et al. (2015) suggested that the increase in effective phosphorus (P_o_ and P_i_) pools through the regulation of microbial biomass and enzyme activities is primarily associated with an overall increase in soil organic matter. In other words, the exogenous inputs of C play a significant role in activating P-cycling microbial abundance. Since the amount of C inputs from different green manure sources in this study were consistent, the effects on functional gene abundances may tend to converge as well (**Figure 5**; **Table 2**).

### 4.3 The growth and P uptake of citrus plants

In P-deficient soil, after the incorporation of green manure residuals, the increase in soil active P pools, along with the enhanced abundance and activity of microorganisms, indirectly promoted the growth, development, and P nutrition status of citrus plants (**Figures 1B, D, F**, **2B, D, F, H**, **6B**; **Table 4**). However, the current research does not provide a consistent conclusion regarding the growth and P nutritional status of citrus plants when different green manure varieties are incorporated. Previous studies (Shackelford et al., 2019; Khan et al., 2022; Ullah et al., 2023) have demonstrated that non-legume green manure incorporation did not favor plant growth, while legume green manure incorporation did. The main reason for the inconsistency observed is that the chemical N supply in this study was sufficient, which may have hindered the beneficial N supply from legume green manure. Secondly, citrus plants are perennial crops and have a slower growth and development compared to annual grain crops. Their slower growth rate and development can limit the observable differences in response to different green manure treatments.

In the P-surplus soils of the present study, the addition of green manure residues did not show a significant improvement in the growth and P nutrition status of citrus plants, except for the P uptake by citrus roots (**Figures 1A, C, E**, **2A, C, E, G**). This unexpected result can be attributed to the adequate availability of pristine soil nutrients and chemical fertilizers and the antagonistic effect of trace element uptake by citrus plants in P-surplus soils. Previous research has consistently demonstrated that high availability of P in the soil can hinder the uptake of essential trace elements such as zinc, copper, and iron by plants (Aboyeji et al., 2020; Singh et al., 2021; Stanton et al., 2022). This interference in trace element uptake can result in reduced yields and overall crop performance in various crops.

### 4.4 Limitations and implications

This study has provided a scientific foundation for quantifying the contributions of green manure to soil P pools and P nutrient of citrus plants, thereby enhancing production efficiency and fostering sustainable development in orchards. However, certain limitations were encountered during the experimental process. Firstly, it is essential to acknowledge that the annual citrus seedlings were employed in this experiment, which differs from the mature citrus trees typically found in actual orchard production. As a result, factors such as the kinetic uptake of soil P and the capacity to regulate the quantity, species, and activity of soil microorganisms vary between seedlings and mature trees. Consequently, the obtained results can only offer limited insights into seedling management within citrus orchards. Secondly, the environmental conditions of the pot experiment were deliberately standardized and controlled, unlike the intricate and dynamic situation present in actual orchard production. The use of potting apparatus may have restricted the growth of plants, particularly impeding root development. Research has demonstrated that P deficiencies induce changes in plant root architecture, leading to an increase in the root-to-shoot ratio, root hairs, root topsoil foraging, and root morphology (Zhang et al., 2013; Su et al., 2014; Iqbal et al., 2020). Hence, in future research endeavors, it is imperative to direct greater attention toward investigating the P uptake status of mature orchards under natural field conditions.

## 5 Conclusion

The present study investigated the influence of diverse green manure species on soil P dynamics and citrus growth across varying initial soil P conditions. The results demonstrated that the introduction of green manure residues positively impacted active P pools in the soil. This effect was attributed to heightened microbial and enzymatic activities involved in P cycling, leading to the conversion of both accumulated soil P and chemical fertilizer P into forms accessible to crops. Notably, legume green manures exhibited superior efficacy in regulating soil P pools, particularly in P-deficient soil. While the addition of green manure residues had limited effects on citrus growth and P uptake in P-surplus soil, its impact was significant in P-deficient soil. Interestingly, there were no substantial differences in the citrus growth and P uptake between legume and non-legume green manure addition treatments. These findings offer valuable insights for fruit farmers seeking to reduce P fertilizer inputs in orchard production, activate insoluble P in soil, mitigate soil P accumulation, and achieve ecological intensification. The study underscores the efficacy of incorporating green manure in orchards as a clean, efficient, and sustainable production model. Importantly, recommending the incorporation of legume green manure, especially in orchards with low P effectiveness, is emphasized. This recommendation aims to enhance P fertilizer utilization, facilitate the conversion of accumulated soil P into active P pools, and promote the overall growth of fruit trees.

## Data availability statement

The raw data supporting the conclusions of this article will be made available by the authors, without undue reservation.

## Author contributions

YY: Data curation, Formal Analysis, Investigation, Methodology, Software, Validation, Visualization, Writing – original draft, Writing – review & editing. WZ: Data curation, Investigation, Methodology, Software, Validation, Visualization, Writing – original draft. XC: Investigation, Writing – original draft. LC: Investigation, Writing – original draft. YL: Investigation, Writing – original draft. QX: Investigation, Writing – original draft. MW: Investigation, Writing – original draft. HY: Investigation, Writing – original draft. RH: Investigation, Writing – original draft. JZ: Investigation, Writing – original draft. YH: Investigation, Writing – original draft. QH: Investigation, Software, Writing – original draft. XS: Funding acquisition, Project administration, Resources, Supervision, Writing – review & editing. YZ: Conceptualization, Funding acquisition, Methodology, Project administration, Resources, Supervision, Visualization, Writing – review & editing.
